# Somatosensory Neuron Typing with High-Coverage Single-Cell RNA Sequencing and Functional Analysis

**DOI:** 10.1007/s12264-017-0147-9

**Published:** 2017-06-13

**Authors:** Changlin Li, Sashuang Wang, Yan Chen, Xu Zhang

**Affiliations:** 1 Institute of Neuroscience and State Key Laboratory of Neuroscience, Center for Excellence in Brain Science, Chinese Academy of Sciences, Shanghai, 200031 China; 2 School of Life Science and Technology, ShanghaiTec University, Shanghai, 200031 China

**Keywords:** Neuron type, Single-cell technology, Somatosensory mechanism, Pain, Gene profiles

## Abstract

Different physical and chemical stimuli are detected by the peripheral sensory receptors of dorsal root ganglion (DRG) neurons, and the generated inputs are transmitted *via* afferent fibers into the central nervous system. The gene expression profiles of DRG neurons contribute to the generation, transmission, and regulation of various somatosensory signals. Recently, the single-cell transcriptomes, cell types, and functional annotations of somatosensory neurons have been studied. In this review, we introduce our classification of DRG neurons based on single-cell RNA-sequencing and functional analyses, and discuss the technical approaches. Moreover, studies on the molecular and cellular mechanisms underlying somatic sensations are discussed.

## Introduction

Peripheral stimuli are detected by the pseudo-unipolar sensory neurons in the dorsal root ganglion (DRG) through molecular sensors at their peripheral nerve terminals. These neurons then transmit peripheral signals to the spinal dorsal horn. Somatosensation comprises different sensory modalities including thermoception, mechanoreception, proprioception, nociception, and pruritoception. There is a notion that different types of DRG neurons are specialized by distinct molecular networks and mediate these sensory modalities. Accumulated experimental evidence has demonstrated that nociceptors mediate other sensory modalities such as mechanoreception [1, 2].

Although the somatosensory molecules have been extensively studied, a global view of the transcriptional profiles of individual DRG neurons has not been obtained. Efforts have been made to understand the gene profiles of these neurons [3–9]. Single-cell RNA-sequencing (scRNA-seq) is an effective technique that has advanced our understanding of single-cell transcriptomes, and the identification of cell clusters of mouse DRG neurons [10, 11]. High-coverage scRNA-seq can provide a better database of single-cell gene profiles [11]. The functions of DRG neurons have been determined by genetic ablation [9, 12]. *In vivo* whole-cell patch recording plus single-cell real-time PCR has been used to determine the cellular functions corresponding to the neuron types [11]. In this review, the scRNA-seq method is discussed. Then, recent studies on the cell types and functional annotations of somatosensory neurons are summarized.

## Methodological Considerations for Neuron-Typing

### Methodological Approach of scRNA-seq

For the isolation of single cells, DRG tissue is subjected to enzymatic digestion and mechanical dissociation. Individual cells are isolated manually or using automated equipment. The microfluidic C1 (Fluidigm) is a commercial device, which automatically performs single-cell capture, reverse-transcription, and cDNA amplification in chip chambers. However, the conditions of neuron dissociation cannot be evaluated by the device but by trained experimenters. To fully control the isolation conditions, manual isolation of neurons is preferred. Under a microscope, healthy DRG neurons have a smooth and bright cell membrane. Individual neurons are picked manually and randomly. Generally, 50 cells can be picked by a trained experimenter within 4 h. The efficiency of manual isolation of single cells is acceptable. The single cell is subjected to reverse transcription and cDNA amplification. The method of scRNA-seq was first reported by Tang in 2009 [13]. Sandberg’s lab introduced Smarter-seq, and the subsequently improved Smarter-seq2 [14, 15]. We used the Smarter-seq commercial kit (Clontech) in our studies to identify the gene profiles of individual neurons.

The sequencing depth is important for obtaining the whole transcriptome of a single neuron. Low-coverage scRNA-seq, which detects 4,644 genes in a representative cell, has been suggested to be sufficient for cell-clustering and biomarker identification [16]. However, this number is much smaller than that by high-coverage sequencing, which detects ~10,000 genes per cell [11]. Moreover, the low-coverage sequencing leads to great variation in genes with transcripts of low or medium abundance [16]. As we attempted not only to identify neuron types and their corresponding biomarkers, but also to reveal the whole transcriptomes of individual neurons, high-coverage scRNA-seq was an appropriate choice in our studies.

The number of sequenced neurons is determined by the minimal number of neurons required to cover all neuron types. More neuron samples are better in the process of identifying neuron types, but this also increases the cost. To minimize the number, a specific approach has been designed according to the cellular properties of DRG neurons. One is that a subpopulation of neurons can be labeled by isolectin B4 (IB4). The other is that neuron size is considered to be correlated with the conventional classification of DRG neurons. In our study, IB4-labeling was shown by IB4-fluorescein while neuron size was determined using a scale within the objective lens. Finally, IB4-negative small neurons (cross-sectional area <800 μm^2^), IB4-positive neurons, and IB4-negative large neurons (cross-sectional area >800 μm^2^) each accounted for ~1/3 of the selected neurons. Individual neurons were collected manually. When the number of sequenced neurons was close to 100, 9 neuron types were identified. Only one more neuron type was identified when the number approached 200. Thus, a number close to 200 is considered to be acceptable.

Recently, the number of publications with scRNA-seq has markedly increased, and various analytical methods have been developed. Based on our experience, we suggest the use of differentially expressed genes, but not all detected genes, for further analysis of cell clusters. Weighted Gene Co-expression Network Analysis is a good method to cluster highly-correlated genes into gene modules [17]. Visualization and cluster analysis of scRNA-seq data can be facilitated by Principal Component Analysis or t-distributed stochastic neighbor embedding (t-SNE) [18]. Technical progress in single-cell capture and the analysis of scRNA-seq data have been reviewed elsewhere [19, 20].

### Evaluation of scRNA-seq Data

Data from scRNA-seq should be evaluated carefully. After bioinformatics analysis, representative genes of a cell or cell type can be identified. By quantitative single-cell RT-PCR, the profiles of multiple genes can be examined in individual cells. *In situ* hybridization (ISH) can be used to analyze the cell distribution of genes in the tissue. However, fluorescent ISH is less sensitive than single-cell RT-PCR. Notably, modified ISH can enhance the sensitivity. A branched DNA ISH has been developed to detect the expression of single-copy genes [21]. However, although branched DNA ISH is very sensitive, the spread of this technique has been limited by competitive interests and the high cost of commercial kits. Recently, a multiplexed fluorescent hybridization chain reaction (HCR) was developed by Pierce’s lab [22]. The cascades of HCR amplify the fluorescent signals. Fluorescent HCR is an alternative, although it is often not sensitive enough to detect gene expression at low levels. Immunostaining of cells is not ideal for the evaluation of scRNA-seq data, because the immunolabeling intensity is not always concordant with the gene expression level [23]. For example, scRNA-seq data show that *Nefh*, which encodes neurofilament 200 (NF200) and is a well-known marker of myelinated DRG neurons, is also expressed in DRG neurons positive for MAS-related GPR family member D (*Mrgprd*). However, previous immunostaining results showed that *Mrgprd*-positive neurons do not contain NF200 [24].

## Somatosensory Neuron Types

DRG neurons are divided into different populations with distinct properties, including small-diameter neurons with unmyelinated C-fibers or thinly-myelinated Aδ-fibers, and large-diameter neurons with thickly-myelinated Aα/β-fibers [25]. The small neurons can be subdivided into two subpopulations: one shows peptidergic characteristics by expressing neuropeptides, substance P and calcitonin gene-related peptide (CGRP) [25]; the other does not express neuropeptides but binds IB4 [25]. In contrast, the large neurons are characterized by the expression of NF200 [25]. Tyrosine hydroxylase (TH) is not expressed in neurons of the three subpopulations above, suggesting that TH-positive neurons comprise another subpopulation [26]. DRG neurons can also be classified by the expression of neurotrophic factor receptors. Peptidergic small neurons express glial-derived neurotrophic factor (GDNF) family receptor α 3 (Gfra3) and ropomyosin receptor-kinase A (TrkA), a nerve growth factor (NGF) receptor. Non-peptidergic small neurons contain Ret receptor and Gfra2. Large neurons express TrkB or TrkC.

Efforts have been made to identify the transcriptional profiles of different subpopulations of DRG neurons. Neuron purification by fluorescent activated cell sorting coupled with gene profiling has been used to characterize the molecular properties of TrkC-positive [7] and TRPV1-positive [6] neurons. Gene profiles of ion channel and GPCRs in trigeminal ganglia and DRGs have been analyzed by RNA-seq [5, 8]. In DRG tissue, glial cells, including satellite glial cells and Schwann cells, outnumber sensory neurons. After magnetic purification, the transcriptomes of the neurons were examined [3]. Recently, Chiu *et al.* [4] used parallel quantitative RT-PCR to analyze the expression levels of candidate genes in single DRG neurons from three subpopulations including IB4^+^ Na_v_1.8-Cre/TdTomato^+^, IB4^−^ Na_v_1.8-Cre/TdTomato^+^, and Pvalb-Cre/TdTomato^+^. The analysis led to the identification of six distinct subgroups, including *Mrgprd* ^+^, *Nppb* ^+^, and *Th* ^+^ neurons [4]. However, these results presented the gene profiles of cell groups, but could not identify the neuron types in an unbiased and integrated manner.

ScRNA-seq is an important technology increasingly used to generate cell atlases of the central [27, 28] and peripheral nervous systems [10, 11]. Usoskin *et al.* reported that peptidergic, non-peptidergic, TH-positive, and NF200-positive mouse DRG neurons can be further classified into 11 subtypes using low-coverage scRNA-seq, which detected ~3,900 ± 1,880 (mean ± SD) genes per neuron [10, 11]. However, the low-coverage scRNA-seq only provided a partial transcriptome, and caused considerable variation in the gene expression profiles, making it difficult to define specific marker genes [10, 11]. In fact, CGRP and NF200 are not good markers for DRG neuron types, because they are expressed in many types [11].

**Fig. 1:**
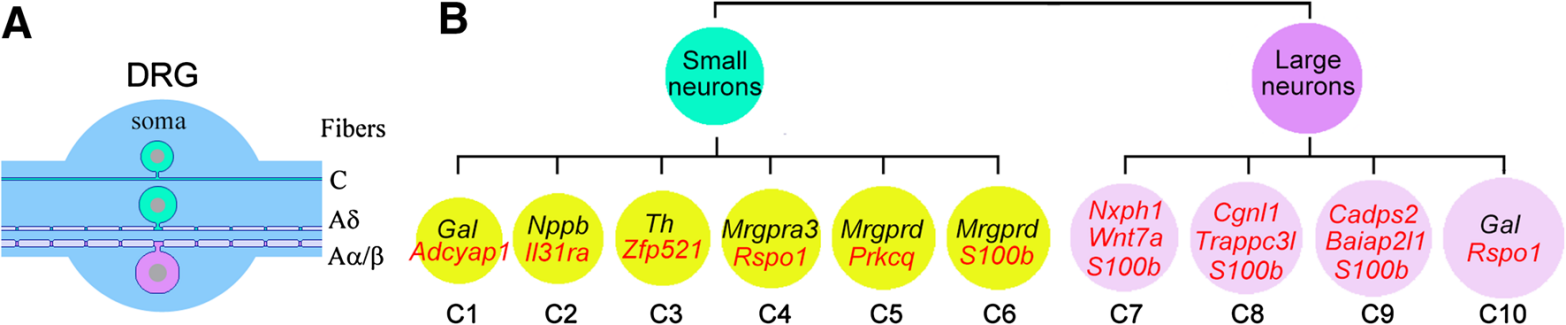
Somatosensory neuron types. **A** morphological characteristics of DRG neurons. **B** Classification of the types and subtypes of DRG neurons, their markers, and the type-hierarchy. New markers are indicated in *red*. (Figure adapted and modified from Li *et al*. [11] with permission).

We performed high-coverage scRNA-seq, which detected 10,950 ± 1,218 genes per neuron, and thus identified 10 types and 14 subtypes of DRG neuron [11]. The profiles are available at our database (http://www.ibrainproject.org/en/index.php?c=channel&a=type&tid=84). We not only provided the transcriptome of individual neurons, but also identified distinct marker genes for the neuron types (C1 – C10) and subtypes (Fig. 1) [11]. Neurons in C1 were marked by galanin (*Gal*), C2 by natriuretic peptide B (*Nppb*), C3 by *Th*, C4 by *Mrgpra3*, C5 by *Mrgprd*, C7 by neurexophilin 1 (*Nxph1*) plus S100 Ca^2+^-binding protein B (*S100b*), C8 by *S100b*, and C9 by BAI1-associated protein 2-like 1 (*Baiap2l1*) plus *S100b*. C6 neurons expressed a high level of *S100b* and a low level of *Mrgprd*. C10 neurons were large neurons expressing *Gal*. Some subtypes were marked by specific functional genes, such as *Mrgprb4* ^+^ in the C4-2 subtype of *Mrgpra3* ^+^ C4, while other subtypes expressed genes representative of myelinated neurons, such as *Nefh* and *S100b* in C2-2 of *Nppb* ^+^ C2 [11]. Thus, the number of neuron types and subtypes identified by high-coverage scRNA-seq [11] is greater than that by low-coverage scRNA-seq [10, 11]. Moreover, the specific markers for each neuron type and subtype can be identified by high-coverage scRNA-seq data.

## Growth Factor Receptors and Somatosensory Neuron Types

The timing-specific coordination of multiple transcriptional factors controls the progress of neurogenesis, differentiation, and specification in sensory neuron lineages [29, 30]. Neurogenin 1 (Ngn1) and Ngn2, basic helix-loop-helix transcription factors, play key roles in the neurogenesis and differentiation of primary sensory neurons. Ngn1 determines the formation of TrkA-positive DRG neurons while Ngn2 regulates the formation of TrkB- and TrkC-positive DRG neurons [29, 30]. Runt-related transcription factor 1 (Runx1) and Runx3 regulate the progress of diversification. Runx3 inhibits the expression of TrkB and contributes to the specification of TrkC-positive DRG neurons. Runx1 is restricted to TrkA-positive DRG neurons before the maturation of non-peptidergic neurons [31]. The maturation of non-peptidergic DRG neurons is controlled by NGF signals, because NGF deficiency leads to a dramatic reduction of *Runx1* and the loss of *Mrgprd* [32]. The maintenance of Runx1 expression results in non-peptidergic neurons by inhibiting the expression of neuropeptides, while the loss of Runx1 leads to peptidergic neurons [29, 30]. Neurotrophin receptors represent four types of DRG neurons: TrkA-positive/Runx1-negative, TrkA-negative/Runx1-positive, TrkC-positive/Runx3-positive, and TrkB-positive. GDNF family receptors include Ret, the common receptor, and Gfrα1–4, its co-receptors. Gfrα1–3 and Ret are expressed in different types of DRG neurons. Ret contributes to the diversity of TrkB-positive neurons [29]. TH is specifically expressed in Gfrα2-positive but neither in Gfrα1- nor Gfrα3-positive DRG neurons [26]. Mrgprd is expressed in Gfra2-positive neurons [33]. Gfrα3 is expressed in small peripherin-positive neurons but not in large NF200-positive neurons [34]. Gfrα3 is predominately co-expressed with TrkA or CGRP in DRG neurons [34].

The distributions of Trk receptors and Gfrα1–3 are closely correlated with DRG neuron types, but how they represent different types is not well determined. Morphological studies could help to define the relationships of TrkA–C and Gfrα1–3 with somatosensory neuron types. However, it is not easy to determine the distributions of these six molecules at the same time. ScRNA-seq technology could meet this demand. According to our scRNA-seq data, *Gal*-positive C1 and C10 neurons highly express *Ntrk1* and *Gfra3*; *Nppb*-positive C2 neurons prefer to express *Ntrk1* or *Gfra3*; *Th*-positive C3 neurons express *Gfra2* but neither *Gfra1* nor *Gfra3*, consistent with the previous report [26]; *Mrgpra3*-positive C4 neurons contain high *Gfra1* and *Ntrk1* but not *Gfra2*; *Mrgprd*-positive C5 and C6 neurons contain *Gfra2* and *Gfra1*; C7 neurons contain *Ntrk3*, while C9 neurons express *Ntrk2*; and C8 comprises two sub-clusters, one containing *Ntrk3* and *Gfra1*, the other expressing high *Ntrk1* (Table 1). Thus, distinct combinations of *Ntrk* and *Gfra* contribute to the discrimination of different DRG neuron types.

**Table 1:** Expression patterns of growth factor receptors in different types and subtypes of DRG neuron

Gene	C1	C2	C3	C4-1	C4-2	C5	C6	C7	C8-1	C8-2	C9	C10
*Ntrk1*	**+++++**	**+++**		**+++**	**+++++**	**+**	**+++**			**+++++**		**+++++**
*Ntrk2*		**+**	**+**			**+**	**+++**		**+++**	**+**	**+++++**	**+++**
*Ntrk3*		**+**	**+++**				**+++**	**+++++**	**+++++**	**+++**	**+++**	
*Ngfr*	**+++**		**+++**	**+**	**+**		**+++**		**+++**	**+++++**	**+++++**	**+++++**
*Gfra1*				**+++++**	**+++++**	**+**	**+++**	**+**	**+++**			
*Gfra2*		**+**	**+++++**			**+++++**	**+++**					
*Gfra3*	**+++++**	**+++**					**+**					**+++++**
*ret*	**+**	**+**	**+++++**	**+++**	**+++**	**+++++**	**+++**		**+++**			**+++**

+++++, high expression; +++, medium expression; +, low expression; blank, lowest expression or not detected

## Identification of Rare Neuron Subtypes

According to our data, 10 types and 14 subtypes of mouse DRG neuron are identifiable. There is always a possibility that a novel type or subtype has not been identified. It has been suggested that the RaceID algorithm can help to identify rare cell types or subtypes within a complex mixture of individual cells [35]. RaceID can identify rare cells expressing outlier genes at levels significantly exceeding the modelled noise [36]. The detailed method was introduced in a report by Grün *et al.* [35]. Using the R script of the RaceID algorithm kindly supplied by the authors, a total of 1745 differentially-expressed genes from 197 DRG neurons subjected to cell clustering in our report [11] were used for RaceID analysis, which classified the 197 neurons into 9 clusters. Cluster 1 was marked by *Mrgprd*, as for the C5 cluster; cluster 2 was marked by secretagogin (*Scgn*) and prokineticin receptor 2 (*Prokr2*), as in C8-2; cluster 3 was marked by *Baiap2l1*, as in C9; however, cluster 4 corresponded to a merger of *Gal*-positive C1, *Nppb*-positive C2, and *Mrgpra3*-positive C4; cluster 5 was marked by *Ntrk3* and *Ptgfr*, as in C8-1; cluster 6 was marked by *Th*, as in C3; and cluster 8 was marked by *Mrgprb4* as in C4-2.

**Fig. 2:**
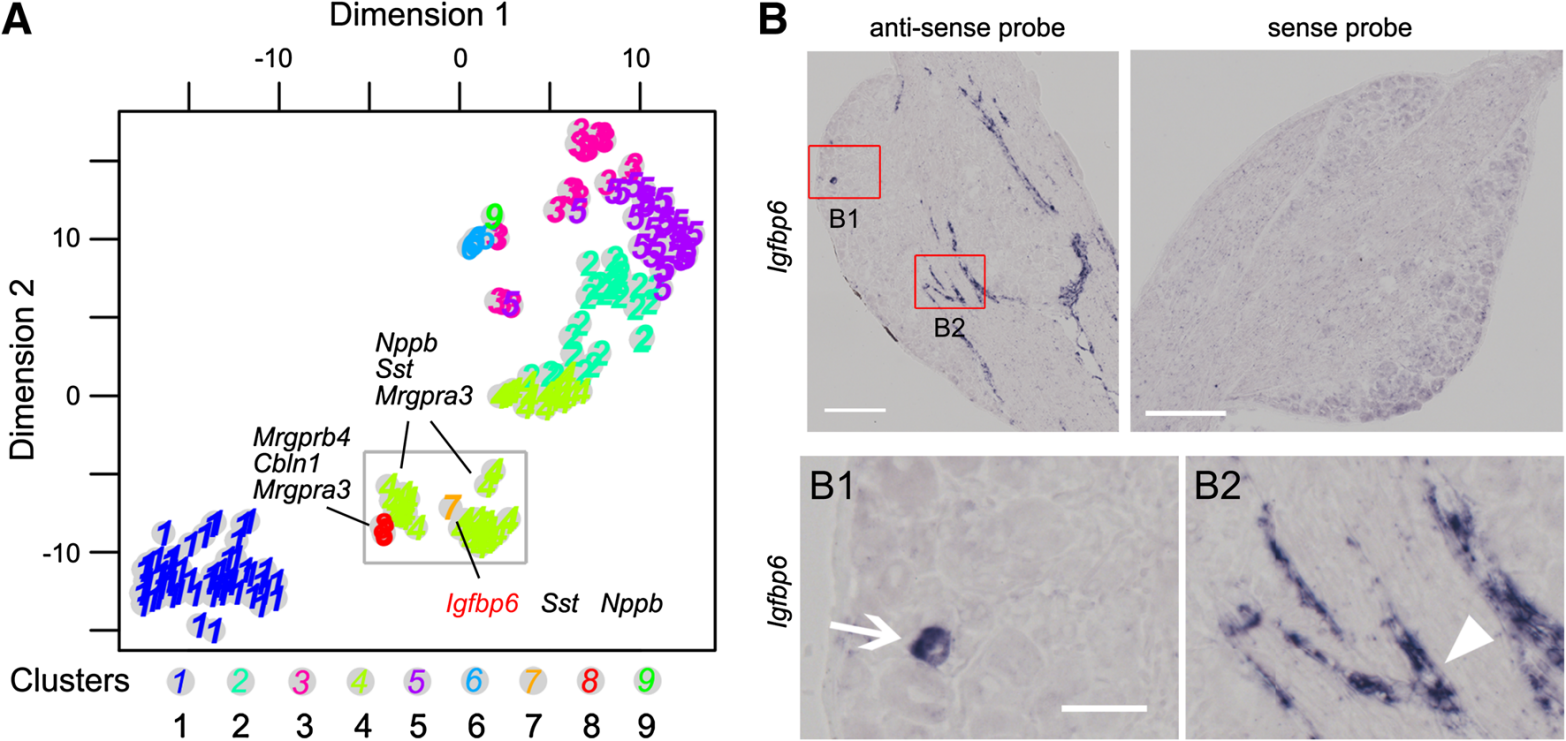
Rare cell types and subtypes of DRG neuron. **A** t-SNE map showing cell clusters obtained using the RaceID algorithm. The *grey square* shows cell cluster 7 and genes corresponding to known markers specifically expressed in the cluster. Cluster numbers are repeated in *black*. **B** Gene expression of *Igfbp6* in mouse DRG shown by ISH with anti-sense probe (*left*). No ISH signal is detected with the sense probe (*right*). Enlargements show the *Igfbp6* expression in a neuron (**B1**, *arrow*) and a fibroblast in a blood vessel (**B2**, *arrowhead*). *Scale bars* for B, 200 μm; for B1 & B2, 50 μm.

Only a single neuron was located in clusters 7 and 9 (Fig. 2A). The neuron in cluster 7 expressed insulin-like growth-factor-binding protein 6 (*Igfbp6*) and *Nppb*, while that in cluster 9 expressed carcinoembryonic antigen-related cell adhesion molecule 10 (*Ceacam10*) and *Th* (Fig. 2A). According to our data, *Ceacam10* was expressed by the majority of *Th*-positive neurons [11], suggesting that *Ceacam10* is a potential marker of *Th*-positive C3 neurons. The neuron of cluster 7 expressed *Igfbp6* with *Nppb*, which is a marker of C2 neurons. *Igfbp6* was only contained in a few *Nppb*-positive C2 neurons. ISH was performed to assess *Igfbp6* expression in the DRG. As reported [37], *Igfbp6* is expressed in endothelial cells, but was rarely found in neuron(s) of mouse DRG (Fig. 2B). The percentage of *Igfbp6*-positive DRG neurons was <1%. The results indicated that *Igfbp6* is a potential marker of a rare subtype of C2 neuron. The function of *Igfbp6*-positive DRG neurons needs to be explored. According to previous reports [10, 11] and the present RaceID analysis, it is unlikely that major types or subtypes of DRG neuron have failed to be identified, although the existence of rare types or subtypes cannot be completely ruled out.

## Multiple Functions of Neuron Types Suggested by Their Transcriptional Profiles

**Fig. 3:**
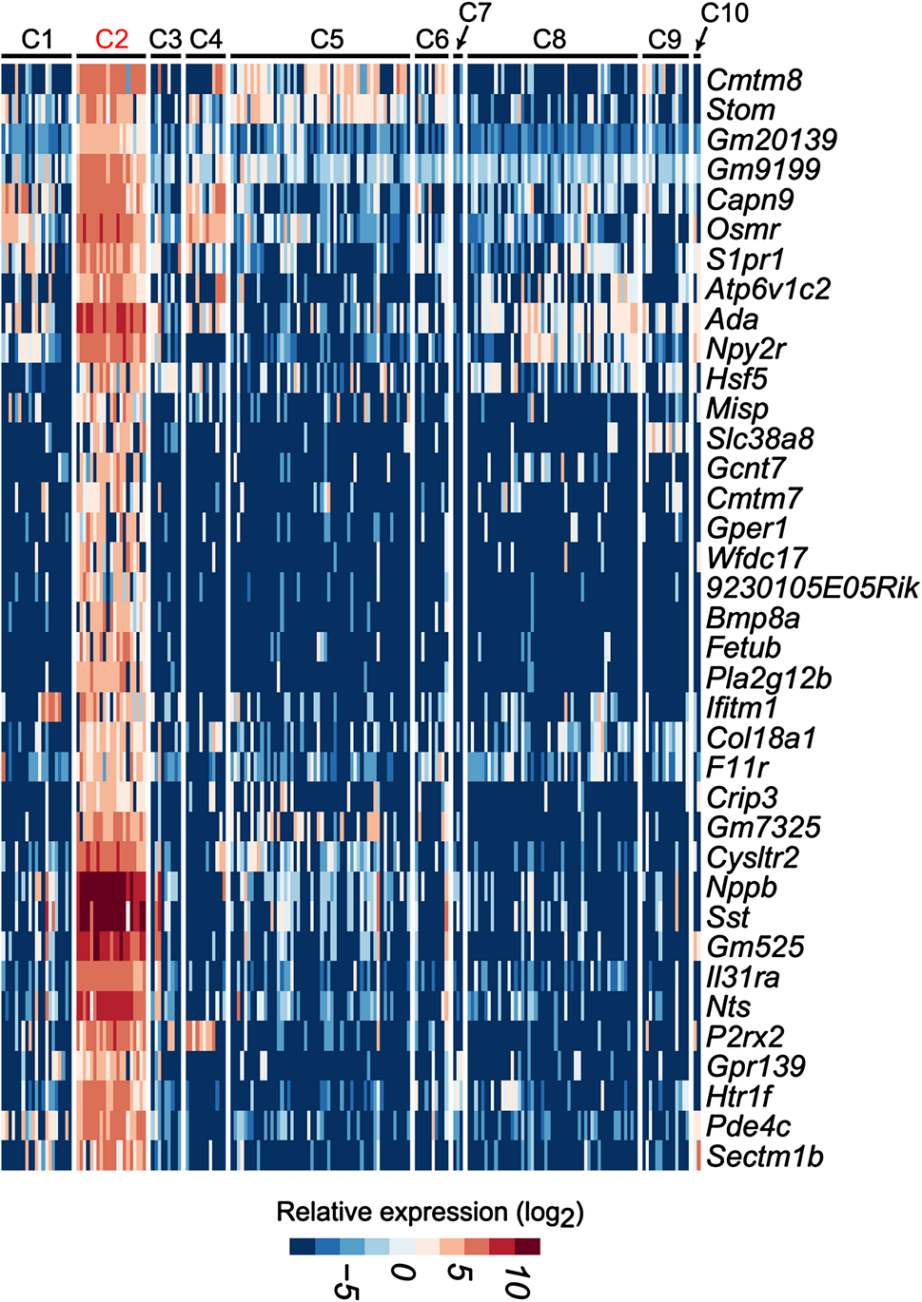
Genes specifically contained in *Nppb*-positive mouse C2 DRG neurons. Heatmap showing the expression patterns of selected genes. (Figure adapted and modified from Li *et al*. [11] with permission).

The mechanisms underlying somatosensory functions have been studied for decades, so at least some resources can be used to predict the possible functions of DRG neuron types. For example, C2 neurons selectively express *Nppb* encoding B-type natriuretic peptide (BNP), *Nts* encoding neurotensin (NTS), *Sst* encoding somatostatin (SST), *Il31ra* encoding interleukin 31 receptor A (Il31ra), and *Htr1f* encoding 5-hydroxytryptamine (serotonin, 5-HT) receptor 1F (*Htr1f*) (Fig. 3) [4, 11]. BNP is involved in the transmission of itch signals [38], while Il31ra mediates T helper cell-dependent itch [39]. Moreover, C2 neurons contain other pruritogen receptors, including *F2rl1*, *Hrh1*, *Htr2a*, and *Mrgprc11*, suggesting that C2 neurons respond to multiple pruritogens [40]. The oncostatin M receptor encoded by *Osmr* forms a functional receptor complex with Il31ra [41], and is expressed specifically in C2 neurons [11]. Serotonin released from the descending pathway might potentiate itch sensation by activating 5-HT1A receptors on dorsal horn neurons [42]. Serotonin also activates 5-HT1A at the central terminals of C2 neurons [11]. Genetic ablation of C2 neurons might reduce itch induced by IL-31 or 5-HT1F agonist [9].

In addition to the role in itch sensation, C2 neurons are involved in nociception. BNP is shown to inhibit spinal nociceptive transmission [43]. The activation of 5-HT1A inhibits nociceptive neurotransmission presynaptically in spinal dorsal horn [44]. *Npy2r* encoding neuropeptide Y receptor 2 is predominantly expressed in small CGRP-positive DRG neurons [45]. In fact, this receptor is expressed in all C2 neurons and a few C1 and C8 neurons [11]. The activation of *Npy2r*-expressing DRG neurons induces abnormally exacerbated pain [46]. Thus, C2 neurons also function in nociceptive neurotransmission.

C2 neurons also specifically express SST, which is co-localized with Il31ra or NTS [10]. Thus, C2 neurons express neuropeptides including BNP, NTS, and SST. Spinal NTS and SST are considered to play inhibitory roles in nociceptive neurotransmission by activating their receptors [47–49]. *Sstr2* encoding a subtype of SST receptor (SSTR2) is present in a subtype of C1 neurons, while *Ntsr2* encoding a subtype of NTS receptor (NTSR2) is found in C1 and C8 neurons [11]. Thus, SST and NTS secreted from C2 neurons might regulate the activity of C1 neurons by acting at SSTR2 and NTSR2, respectively.

## Functional Annotation of DRG Neuron Types and Subtypes

We propose that neuron types should be defined by integrating the transcriptomic, morphological or anatomical, and functional characteristics of neurons. The morphological or anatomical characteristics of neuron types include the sizes of their cell bodies, axons and dendrites, and inputs and outputs of circuits. To find the functions of neuron types, we performed *in vivo* whole-cell patch recordings to determine the neuronal responses to peripheral stimuli, and then carried out single-cell real-time PCR to identify the neuron type [11]. We found that most types of nociceptors respond to multiple stimulus modalities and others have more specialized response properties. Sensing of noxious heat and mechanical stimuli may be the principal function of many types (C1, C2, C4, C5, and C6) and subtypes of small DRG neurons (mechanoheat nociceptors) that could also be specialized for other somatosensory properties such as itch [11]. Two types of nociceptors more specialized for noxious mechanical stimuli have been found in large DRG neurons (C7 and C9) [11]. Most mechanoheat nociceptors are also polymodal nociceptors because they are also sensitive to various chemical stimuli. The TH-positive C3 neurons function as C-LTMRs (low-threshold mechanoreceptors), and C8 neurons differentially express *Trpc1*, *Kcnk4* encoding K^+^ channel, subfamily K, member 4, *Asic3* encoding acid-sensing (proton-gated) ion channel 3, and *Piezo2* encoding piezo-type mechanosensitive ion channel component 2, suggesting roles in mechanoreception. C3 and C8 are two unique neuron types that may serve as mechanoceptors.

It is interesting to further analyze how the particular somatosensory functions are mediated by the gene networks in the neuron types and subtypes. For example, we asked whether all types of mechanoheat nociceptors have the same mechanism of mediating noxious heat sensation. In the past decades, intense research led to the discovery of the mechanisms underlying thermal nociception. Although TRPV1 has been proposed to play an important role in heat nociception, *Trpv1*-knockout mice only show a partial deficit in noxious heat assays [50]. Our study showed that the majority of identified types of small DRG neurons respond to noxious heat stimuli, while *Trpv1* is present in C1 and C2 neurons [11]. Importantly, we have found a critical role of fibroblast growth factor 13 (FGF13) in heat nociception. FGF13 is highly expressed in C1, C2, C4, C5, and C6 DRG neurons, and these were identified as mechanoheat nociceptors. Heat nociception, but not mechanical nociception, is completely abolished in *Fgf13*-deficient mice [51]. One related mechanism is that FGF13 interacts with Na_v_1.7 and increases its currents. *Fgf13*-deficiency causes a reduction of Na_v_1.7 in the plasma membrane and therefore markedly reduces the action potential firing during noxious heat stimulation. Patients with loss-of-function mutations of Na_v_1.7 show the congenital absence of pain perception [52]. Thus, the FGF13/Na_v_1.7 interaction may act as a common and final regulatory pathway for most types of mechanoheat nociceptors to transmit noxious heat signaling.

## Conclusions and Perspectives

This review underscores the importance of high-coverage scRNA-seq in determining the single-cell transcriptome by discussing our studies using scRNA-seq, analysis of DRG neuron types, and their corresponding functional annotation. High-coverage scRNA-seq can reveal most of the gene expression profile of a single neuron. Functional analysis provides a framework for the somatosensory functions of neuron types and subtypes. However, the gene network mechanisms underlying various somatic sensations remain to be further investigated. The neuron type-specific circuits that transmit sensory information into the central nervous system need to be demonstrated. Moreover, the functions corresponding to different types of DRG neurons should be studied in detail using multiple experimental approaches, including *in vivo* patch recording, optogenetic techniques, and chemical genetic methods.
